# Isotope Composition and Chemical Species of Monthly Precipitation Collected at the Site of a Fusion Test Facility in Japan

**DOI:** 10.3390/ijerph16203883

**Published:** 2019-10-14

**Authors:** Naofumi Akata, Masahiro Tanaka, Chie Iwata, Akemi Kato, Miki Nakada, Tibor Kovács, Hideki Kakiuchi

**Affiliations:** 1 Department of Radiation Chemistry, Institute of Radiation Emergency Medicine, Hirosaki University, Hirosaki 036-8564, Japan; 2 Department of Helical Plasma Research, National Institute for Fusion Science, National Institutes of Natural Sciences, Toki 509-5292, Gifu, Japan; tanaka.masahiro@nifs.ac.jp; 3 Department of Engineering and Technical Services, National Institute for Fusion Science, National Institutes of Natural Sciences, Toki 509-5292, Gifu, Japan; iwata.chie@nifs.ac.jp (C.I.); kakemi@nifs.ac.jp (A.K.); nakada.miki@nifs.ac.jp (M.N.); 4 Institute of Radiochemistry and Radioecology, University of Pannonia, H-820010 Egyetem Str, Veszprém, Hungary; kt@almos.uni-pannon.hu; 5 Department of Radioecology, Institute for Environmental Sciences, Aomori 039-3212, Japan; ckhsd@ies.or.jp

**Keywords:** tritium monitoring, fusion test facility, deuterium plasma experiment, monthly precipitation, chemical composition

## Abstract

The deuterium plasma experiment was started using the Large Helical Device (LHD) at the National Institute for Fusion Science (NIFS) in March 2017 to investigate high-temperature plasma physics and the hydrogen isotope effects towards the realization of fusion energy. In order to clarify any experimental impacts on precipitation, precipitation has been collected at the NIFS site since November 2013 as a means to assess the relationship between isotope composition and chemical species in precipitation containing tritium. The tritium concentration ranged from 0.10 to 0.61 Bq L^−1^ and was high in spring and low in summer. The stable isotope composition and the chemical species were unchanged before and after the deuterium plasma experiment. Additionally, the tritium concentration after starting the deuterium plasma experiment was within three sigma of the average tritium concentration before the deuterium plasma experiment. These results suggested that there was no impact by tritium on the environment surrounding the fusion test facility.

## 1. Introduction

The sources of environmental tritium (^3^H), a radioisotope of hydrogen that decays to ^3^He with a half-life of 12.3 years, have been summarized by researchers [1,2,3]. Most naturally sourced tritium is produced by the interaction of cosmic rays with nitrogen (^14^N) and oxygen (^16^O) atoms in the upper atmosphere. The tritium production rate by cosmic rays is estimated as 0.25 atoms cm^−2^ s^−1^ [2]. The global production rate of natural tritium is 72 × 10^15^ Bq y^−1^ if it is assumed that surface area of the earth is 5.1 × 10^14^ m^2^. On the other hand, anthropogenic ^3^H arises from several sources. Atmospheric nuclear weapon testing from the 1950s to the early 1960s released significant amounts of tritium into the environment [2], and approximately 1.86 × 10^20^ Bq (650 kg) of tritium was released during 1945 to 1985 [4]. Tritium concentrations in precipitation were rapidly increased by these events, and many researchers found high tritium concentrations in precipitation [5,6]. Even now, residual artificial tritium is estimated to be approximately 10^18^ Bq. Nuclear facilities such as nuclear power reactors and nuclear fuel reprocessing plants also release tritium to the environment, and they have become the dominant anthropogenic source. Annual average amounts of released tritium from nuclear facilities worldwide to the atmosphere were estimated to be 11.7 × 10^15^ Bq during the period from 1998 to 2002 [4]. This amount corresponds to approximately 15 to 20% of the annual tritium production rate by cosmic rays. The amount of tritium on the earth was estimated at approximately 1.0–1.3 × 10^18^ Bq and is dependent on the balance between production rate and radioactive decay rate. Accident-released tritium is also important for the natural environment. After the Chernobyl Nuclear Power Plant accident and the Fukushima Dai-ichi Nuclear Power Plant accident, elevated tritium in environmental samples was observed [7,8]. In the case of Japan, Nakasone et al. [9] concluded that tritium concentrations in monthly precipitation were increased by nuclear weapon testing and the Fukushima Dai-ichi Nuclear Power Plant accident. In the future, nuclear fusion reactors will have a large inventory of tritium as fuel. The fuel of nuclear fusion reactors would be the hydrogen isotopes, deuterium (D) and tritium (T). In the 1990s, the Tokamak Fusion Test Reactor (TFTR) in New Jersey, USA and the Joint European Torus (JET) in Oxfordshire, UK were used to carry out D–T plasma experiments. Although the inventory of tritium in these facilities was less than 10 g and tritium in the facilities was confined using a safety system, tritium concentrations in their surrounding environments were slightly increased [10]. The International Thermonuclear Experimental Reactor (ITER) is another international nuclear fusion research and engineering project, and a large experimental tokamak nuclear fusion device designed to study fusion plasmas of D–T reaction is now under construction in Saint-Paul-lès-Durance, France [11]. It is envisioned to be the next major step in the world’s fusion programs. Within the ITER, a total inventory of about 2 to 3 kg (10^18^ Bq) will be necessary to implement the D–T reaction [12]. Therefore, it is important to understand the tritium level in a surrounding environment before and after facility operation starts [13].

The Large Helical Device (LHD) was constructed by the National Institute for Fusion Science (NIFS) at Toki City, Gifu Prefecture, and it is one of the world’s largest magnetically confined helical type fusion experimental devices [14]. The deuterium plasma experiment using LHD was started there in March 2017 to investigate high-temperature plasma physics and the hydrogen isotope effects [14]. A small amount of tritium was produced by a fusion reaction in the deuterium plasma experiment. Although the deuterium and the tritium gases were exhausted from the vacuum vessel of the LHD and recovered by the exhaust detritiation system (EDS) [15], a part of the tritium was released into the environment through the main stack. In the first year of the deuterium plasma experiment, the annual tritium yield was permitted up to 3.7 × 10^10^ Bq for commissioning of the deuterium plasma experiment. The amount of tritium released from the stack was monitored, and the total amount of tritium released during the first year of the deuterium plasma experiment (from 6 March 2017 to 31 March 2018) was approximately 0.13 × 10^9^ Bq [16]. This value was negligibly small compared with the permitted annual tritium yield (3.7 × 10^10^ Bq). The tritium concentrations in environmental samples (environmental water, air, vegetation, etc.) were monitored before and after the deuterium plasma experiment to assess the impact of released tritium [17,18,19,20,21]. The tritium concentration levels were within the background range of the environmental variation. As part of the environmental tritium monitoring, tritium concentration and chemical composition in monthly precipitation and radioactive materials in monthly total deposition samples were monitored.

In order to clarify the experimental impact on precipitation, precipitation has been collected at the NIFS site in Toki, Japan since November 2013 as a means to assess the relationship between isotope composition and chemical species in precipitation with tritium concentration. Partial data of tritium concentration and stable isotope ratio in precipitation before the deuterium plasma experiment have already been reported [21]. This paper reports isotope composition and chemical characteristics of monthly precipitation collected at Toki and discusses the impact of the first year of the deuterium plasma experiment in the LHD on the surrounding environment.

## 2. Materials and Methods 

### 2.1. Overview of Study Site

**Figure 1:**
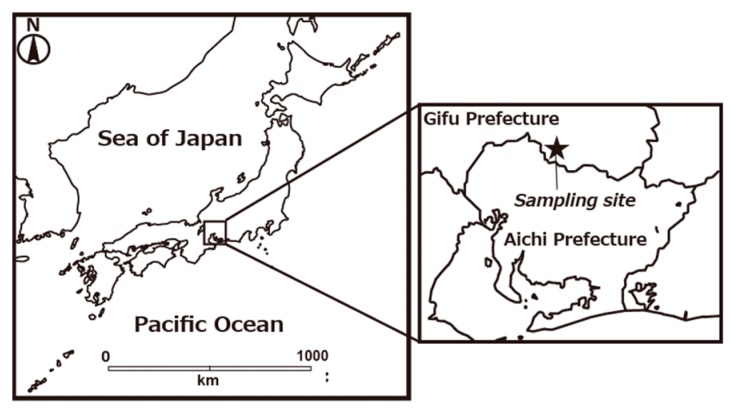
Maps showing location of the sampling site.

The study sampling site was on the roof of a building at the NIFS at Toki City, Gifu Prefecture (35°19′ N, 137°10′ E). Figure 1 shows the sampling location. Toki City is located approximately 30 km northeast of Nagoya City in the central region of Japan. Toki is located in a small-scale basin, approximately 10 km in diameter, and it is surrounded by low elevation mountains. The Meteorological Agency has reported average weather conditions for 30 years (1981–2010) observed at the Tajimi AMeDAS (Automated Meteorological Data Acquisition System) site, which is located 6 km northwest of NIFS [22]. The average monthly precipitation was high in summer and low in winter, while the average wind speed was lower than 1.0 m s^−1^ in that time period. Average monthly temperature ranged from 2.9 to 27.4 °C [23].

### 2.2. Sample Collection and Analysis

Monthly precipitation was collected at NIFS. Precipitation samples were collected from November 2013 to the end of 2017 using a precipitation sampler (ST-1F, Suntechno, Tokyo, Japan) with a 10 L polyethylene container that had been washed with pure water. After measuring sample weight, pH (B-211, Horiba, Kyoto, Japan) and electrical conductivity (EC) (E-771, Horiba) were measured. Approximately 1 L of sample water was distilled, and 800 mL of distilled sample water was electrolyzed to a volume of 65 mL to enrich its tritium content using an electrolytic enrichment system with a solid polymer electrolyte membrane (XZ001, De Nora Permelec Ltd. Fujisawa, Japan). After distilling the tritium-enriched sample water, 50 mL of sample water was mixed with the same volume of a liquid scintillation cocktail (Ultima Gold LLT, PerkinElmer, Waltham, MA, USA) in a 145 mL low diffusion polyethylene vial with an inner Teflon coating. Tritium radioactivity was measured with a low background liquid scintillation counter (LSC: LSC-LB5 or LSC-LB7, Hitachi, Tokyo, Japan) for 1500 min. Counting efficiencies were determined using standard tritium solution (SRM 4361C, NIST, Gaithersburg, MD, USA). The minimum detection level (MDL) of LSC-LB5 and LSC-LB7 with the electrolytic enrichment system was approximately 0.04 Bq L^−1^. Measured values were corrected for radioactive decay to the middle of the sampling period [21].

A part of each sample was filtered using a 0.45 μm membrane filter (DISMIC 25CS045AS, ADVANTEC, Tokyo, Japan). The ionic species (Cl^−^, NO_3_^−^, SO_4_^2−^, Na^+^, Mg^2+^, K^+^, Ca^2+^, NH_4_^+^) in the filtered samples were determined by ion chromatography (ICS-2100, Dionex, Sunnyvale, CA, USA). The Gard column and the Separation column produced by Dionex Inc. were used Ion Pac AG17 4 × 50 mm and Ion Pac AS19 4 × 250 mm for anion analysis and Ion Pac CG16 5 × 50 mm and Ion Pac CS16 5 × 250 mm for cation analysis. We used anion mixed standard solution and cation mixed standard solution (Kanto Chemical Co. Inc. Tokyo, Japan) for quality control. Stable isotope analysis was performed using both an isotope ratio mass spectrometer (Delta V Advantage, Thermo Fisher Scientific, Waltham, MA, USA) with a water equilibrium device (Nakano Electric Inc. Kyoto, Japan) and a cavity ring-down spectroscopy isotopic water analyzer (model L1102-i, Picarro Inc. Sunnyvale, CA, USA) with a CTC analytics autosampler (HTC-PAL, Leap Technologies, Carrboro, NC, USA). Measurement precision was better than ± 1.5‰ for δD and ± 0.15‰ for δ^18^O.

## 3. Results and Discussion

### 3.1. Tritium Concentration in Precipitation

**Figure 2:**
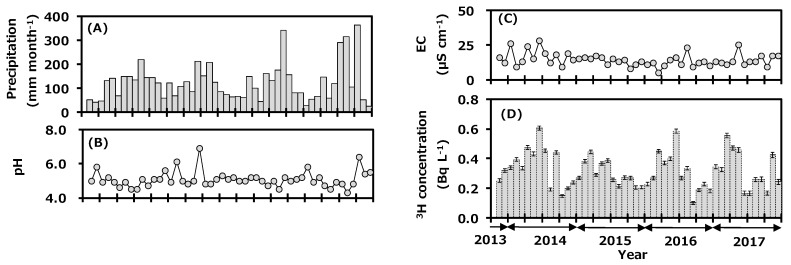
Monthly variations of precipitation amount (**A**), pH (**B**), electrical conductivity (EC) (**C**), and tritium concentration (**D**) in precipitation collected at Toki, Japan before (2013–2016) and after (2017) the first campaign of the deuterium plasma experiment.

Figure 2 shows the monthly variations of precipitation data collected at Toki during the sampling period; they are precipitation amount (A), pH (B), EC (C), and tritium concentration (D). Monthly precipitation amount ranged from 26 to 363 mm and was high in summer and low in winter. Annual precipitation amounts in 2014, 2015, 2016, and 2017 were 1515, 1435, 1550, and 1626 mm with the arithmetic mean value of 1532 mm. Average annual precipitation amount for 30 years (1981–2010) was 1626.7 mm [22], and the value in 2017 was comparable to the reported value, while those of 2014, 2015, and 2016 were slightly lower than the reported values. The pH data ranged from 4.3 to 6.9, and about 56% of the samples were in the range of acid rain with the pH < 5.0. The EC ranged from 5 to 28 μS cm^−1^. There was no clear seasonal trend for either pH or EC.

The tritium concentration in the monthly precipitation ranged from 0.10 to 0.61 Bq L^−1^ and was high in spring and low in summer. Annual tritium concentrations (arithmetic mean ± standard deviation) in 2014, 2015, 2016, and 2017 were 0.35 ± 0.14, 0.30 ± 0.08, 0.30 ± 0.13, and 0.32 ± 0.13 Bq L^−1^. Recent data for tritium concentration in monthly precipitation in Japan have been summarized [9,24,25]. It is known that the concentration of environmental tritium depends on the latitude of the sampling location; it is high at northern latitudes and low at southern latitudes [26,27]. For example, tritium concentration in monthly precipitation in Sapporo (northern Japan) during July 2015 to December 2017 ranged from 0.24 to 1.27 Bq L^−1^ [9], that of Rokkasho during April 2001 to March 2006 ranged from 0.16 to 1.23 Bq L^−1^ [28], that of Chiba during November 2013 to December 2017 ranged from 0.12 to 0.53 Bq L^−1^ [24], and that of Okinawa (southern Japan) during June 2014 to December 2017 ranged from 0.05 to 0.27 Bq L^−1^ [9]. The results of the present study were similar to those of Chiba, which is at a similar latitude. The seasonal trend for tritium concentration in precipitation was similar to the general background pattern observed in Japan, which is high in spring and low in summer [27]. The northwestern monsoon from the Asian continent blows into Japan during winter to spring. A relatively higher tritium concentration in precipitation was observed in inland continental areas due to recycling of tritium by evaporation and precipitation, the so-called continental effect [29]. On the other hand, high-pressure systems develop in the Pacific Ocean in summer and bring air masses to Japan from the ocean, which have slightly lower tritium concentration [28]. Tritium concentration in precipitation at Toki changed with meteorological conditions depending on the air-mass transportation course. The transfer of cosmogenic tritium from the upper atmosphere to the troposphere in spring also seemed to contribute to the seasonal pattern observed.

### 3.2. Stable hydrogen and Oxygen Isotope Composition in Precipitation

**Figure 3:**
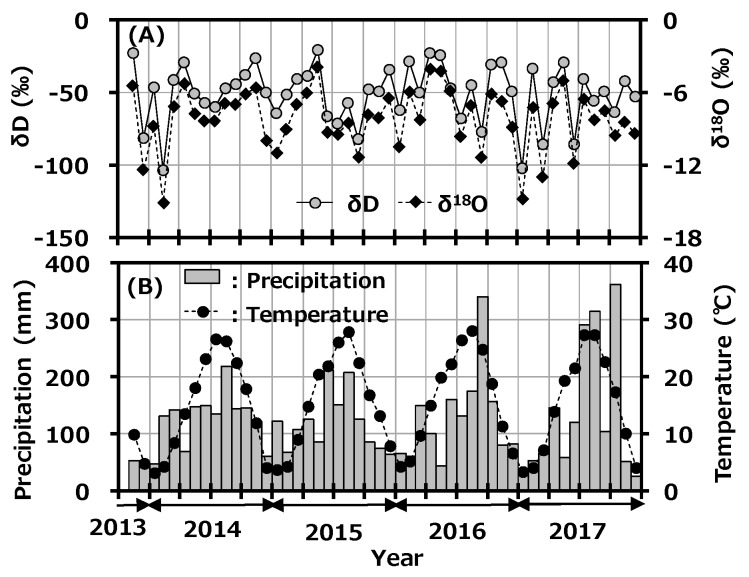
Variations of δD and δ^18^O in monthly precipitation collected at Toki, Japan with monthly precipitation amount (**A**) and the reported monthly average temperature at the Tajimi AMeDas site (**B**).

The seasonal variations of δD and δ^18^O in monthly precipitation at Toki are shown in Figure 3A with the monthly precipitation amount, and the reported monthly average temperature at Tajimi AMeDas site [22] is shown in Figure 3B. δD and δ^18^O in monthly precipitation ranged from −103.62 to −20.77‰ and −15.14 to −3.92‰, respectively. Although precipitation amount and average temperature had a clear seasonal trend, which was high in summer and low in winter, there was no clear seasonal change in δD and δ^18^O. A weak seasonal change of δD and δ^18^O in precipitation was reported to have been observed in the East Asian region [30]. The present results had a similar seasonal trend to this reported one.

In general, δD and δ^18^O in global precipitation are well-related by the following equation.

(1) $$dD\;=\;8.0\;\times \;\delta^{18}O\;+\;10$$

**Figure 4:**
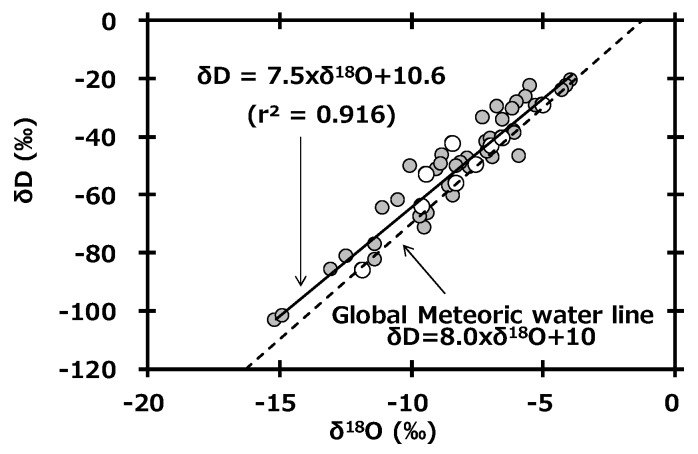
Relationship between δD and δ^18^O in monthly precipitation collected at Toki, Japan and a plot of the global meteoric water line.

This equation, called the global meteoric water line (GMWL), is based on precipitation data collected worldwide [31]. Figure 4 shows the relationship between δD and δ^18^O in monthly precipitation collected at Toki, Japan. The slope of the regression line (δD = 7.5 × δ^18^O + 10.6) of precipitation at Toki was similar to the slope of the GMWL. Additionally, the reported equation based on observations in the Kanto area of Japan (Kumagaya, Saitama: δD = 7.4 × δ^18^O + 9.6, r^2^ = 0.868) [32] was also similar to our equation. Here, the intercept of GMWL is known as the deuterium excess (d-excess), and its equation is as follows [33].

(2) $$d-excess\;=\delta D\;-\;8\;\times \;d^{18}O$$

**Figure 5:**
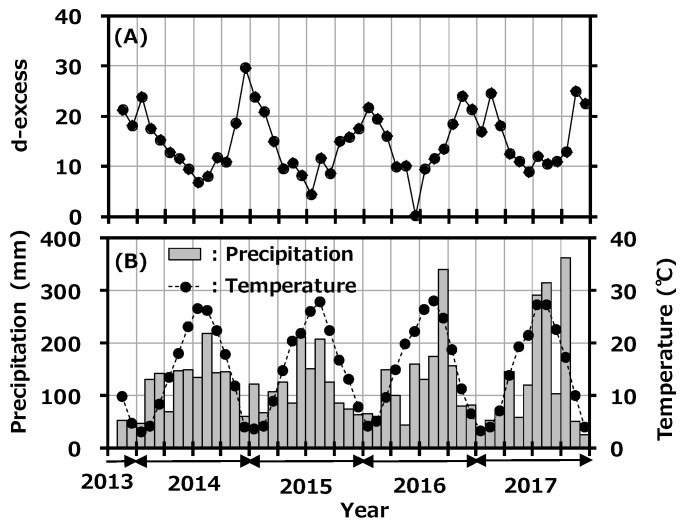
Variation of deuterium excess (d-excess) in monthly precipitation collected at Toki, Japan with monthly precipitation amount (**A**) and the reported monthly average temperature at the Tajimi Automated Meteorological Data Acquisition System (AMeDas) site (**B**).

The d-excess value is used as a convenient tool for describing conditions affecting evaporation in oceanic moisture source regions. There have been some reports about seasonality of d-excess in precipitation in Japan [34,35]. In general, high d-excess indicates lower relative humidity in the maritime air-mass source region [36]. Figure 5A shows the variation of d-excess in monthly precipitation collected at Toki with precipitation rate, and Figure 5B shows the reported monthly average temperature at the Tajimi AMeDas site [22]. The d-excess had clear seasonal variation and was high in winter and low in summer; d-excess and average temperature were negatively correlated (r^2^ = 0.647). As mentioned before, the northwestern monsoon from the Asian continent blows onto the Japanese islands during winter to early spring and carries a dry air-mass with water vapor evaporated rapidly from the Japan Sea. On the other hand, high-pressure systems develop in the Pacific Ocean in summer and bring air-masses from the southern maritime area. This seasonal variation is a general trend seen in Japan, and the present d-excess value was comparable to the reported value [35]. No impact was found from the deuterium plasma experiment on the environment.

### 3.3. Ion Concentrations in Precipitation

**Figure 6:**
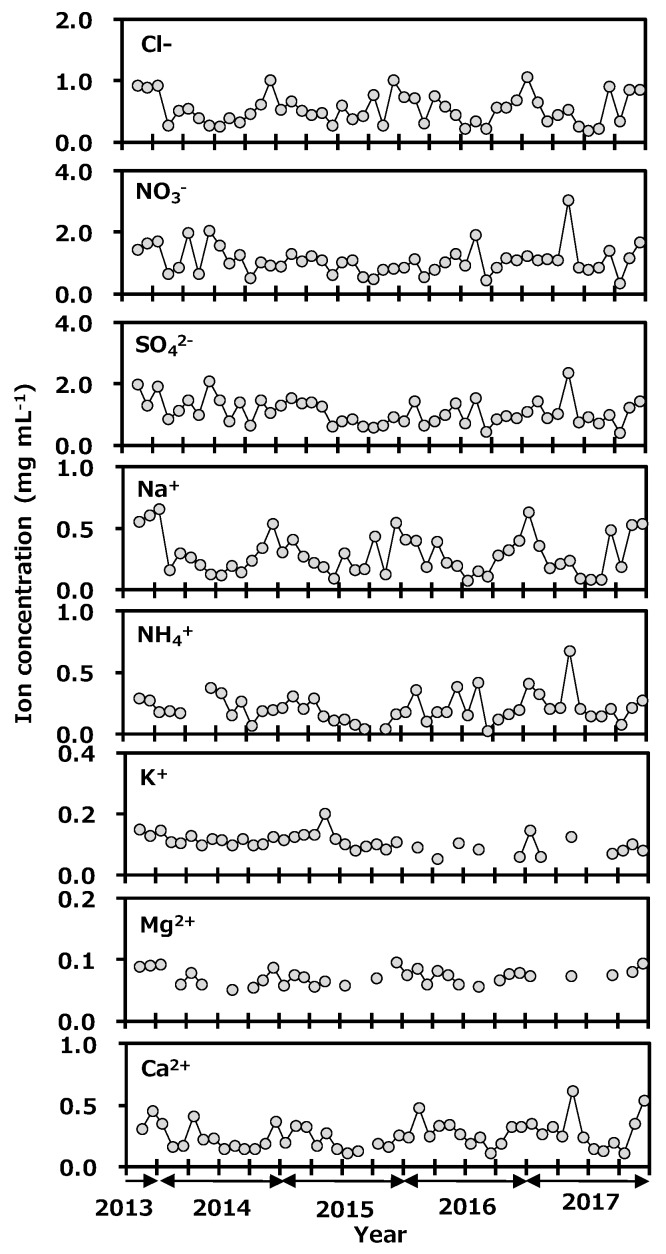
Variation of anion and cation concentrations in monthly precipitation collected at Toki, Japan.

Concentrations of anions and cations in precipitation collected at Toki are shown in Figure 6. Concentration ranges of the anions were Cl^−^, 0.19 to 1.06 mg L^−1^; NO_3_^−^, 0.35 to 3.06 mg L^−1^; and SO_4_^2−^, 0.41 to 2.35 mg L^−1^. The ranges of cations were Na^+^, 0.08 to 0.66 mg L^−1^; NH_4_^+^, 0.03 to 0.68 mg L^−1^; K^+^, <0.05 to 0.20 mg L^−1^; Mg^2+^, <0.05 to 0.10 mg L^−1^; and Ca^2+^, 0.11 to 0.62 mg L^−1^. Seasonal variations of Cl^−^, Na^+^, and Mg^2+^ were a high concentration in winter and a low one in summer. Those of NO_3_^−^, SO_4_^2−^, and NH_4_^+^ were a high concentration in spring and a low one in other seasons. The correlation coefficients of ion concentrations in monthly precipitation at Toki, Japan are shown in Table 1. The relationship between Na^+^ and Cl^−^ concentrations was found to have a strong correlation (*r* = 0.97, *p* < 0.01), and the Na^+^/Cl^−^ concentration ratio was similar to their ratio in seawater, and Mg^2+^ and Cl^−^ also showed a good correlation (*r* = 0.81, *p* < 0.01). It was suggested that Na^+^, Cl^−^ and Mg^2+^ originated from seawater. A good correlation was found between NO_3_^−^, SO_4_^2−^, and NH_4_^+^ (*r* > 0.80, *p* < 0.01). It was reported that main anthropogenic sources of NH_4_NO_3_ are biomass burning, fossil fuel combustion, and gas to particle conversion, and those of (NH_4_)_2_SO_4_ are biomass burning, fossil fuel combustion, vehicle exhaust, and gas to particle conversion [37]. In spring, the continental air-mass is coming to Japan. The present species concentration results indicated that NO_3_^−^, SO_4_^2−^, and NH_4_^+^ were transported from the Asian continent to Japan as long-range transported pollutants. In May 2017, NO_3_^−^, SO_4_^2−^, NH_4_^+^, and Ca^2+^ were seen to rapidly increase. The Japan Meteorological Agency reported that a large-scale Asian dust event known as *kosa* was observed on the 7th and the 8th of May 2017 near the sampling site [38]. From these results, NO_3_^−^, SO_4_^2−^, and NH_4_^+^ were thought to have been transported by the *kosa* in the chemical forms of NH_4_NO_3_ and (NH_4_)_2_SO_4_. It seemed that there was no impact from the deuterium plasma experiment on the chemical species in the precipitation.

### 3.4. Environmental Assessment of Impact from the Deuterium Plasma Experiment

After starting the first campaign of the deuterium plasma experiment in April 2017, a part of the produced tritium was released into the atmosphere through the facility stack [16]. Here, the impact of the experiment on monthly precipitation is discussed. After starting the deuterium plasma experiment, tritium concentration in precipitation ranged from 0.17 to 0.47 Bq L^−1^, which was similar to the concentration range before the experiment, and the seasonal variation was similar too (Figure 2). All data were within three sigma of the average tritium concentration before the deuterium plasma experiment [21]. From this result, it was suggested that there was no impact by tritium on the surrounding environment of the fusion test facility.

**Figure 7:**
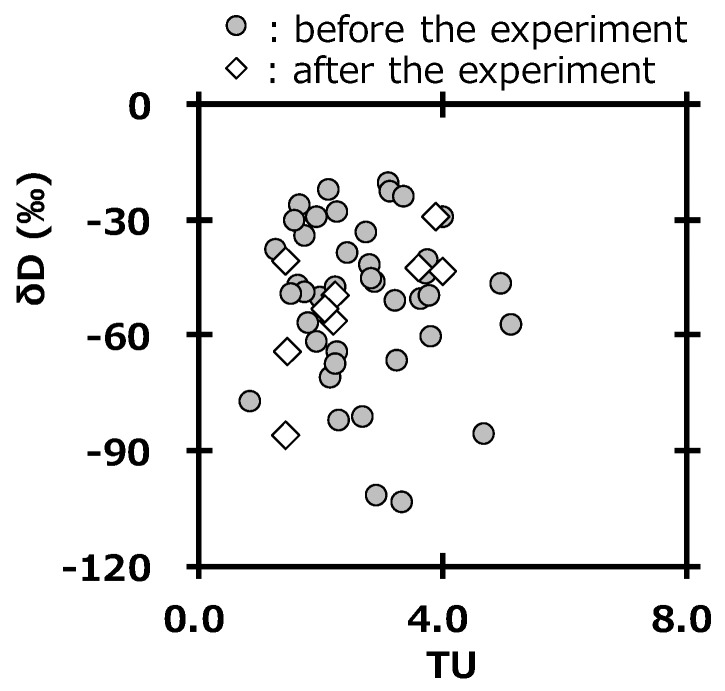
Relationship between tritium concentration (TU) and δD in precipitation at Toki.

The survey of deuterium in precipitation is also important to assess the impact from the deuterium plasma experiment, because deuterium gas is used as fuel gas [21]. The unit of tritium concentration usually used is the tritium unit (1 TU = 0.118 Bq L^−1^). Figure 7 shows the relationship between tritium concentration (TU) and δD in precipitation at Toki. The values were in the same area of the graph before and after the deuterium plasma experiment. This also suggested that there were no impacts by tritium and deuterium on the surrounding environment of the fusion test facility after the deuterium plasma experiment. Additionally, these results supported those reported by Tanaka et al [20]. A committed effective dose equivalent of 6.2 × 10^−6^ mSv y^−1^ was estimated for an annual consumption of drinking water having the highest tritium concentration in precipitation after starting the deuterium plasma experiment (0.47 Bq L^−1^) by using a dose conversion factor of 1.8 × 10^−11^ Sv/Bq [39] and a daily water intake rate of 2.0 L [40]. This value was negligibly small compared with 1 mSv, which is the index of the annual dose limit for the general public. 

## 4. Conclusions

The deuterium plasma experiment using the LHD was started in March 2017 to investigate high-temperature plasma physics and the hydrogen isotope effect. Although deuterium and tritium gas were exhausted from the vacuum vessel of the LHD and recovered by the exhaust detritiation system, a small amount of hydrogen gas was released into the atmosphere through the main stack. To assess the impact of released tritium to precipitation, monthly precipitation had been monitored at the NIFS site from November 2013 to establish background values for tritium, hydrogen, and oxygen stable isotope compositions and chemical species. The tritium concentration ranged from 0.10 to 0.61 Bq L^−1^ and was high in spring and low in summer. δD and δ^18^O in monthly precipitation ranged from −103.62 to −20.77‰ and −15.14 to −3.92‰, respectively. There was no clear seasonal change in δD and δ^18^O, and the findings were similar to reported seasonal data. The slope of the line showing the relationship between δD and δ^18^O in monthly precipitation (δD = 7.5 × δ^18^O + 10.6) was similar to the slope of GMWL. The d-excess showed clear seasonal variation, which was high in winter and low in summer; d-excess and average temperature were negatively correlated (r^2^ = 0.647). As a result of ion species measurements, NO_3_^−^, SO_4_^2−^, and NH_4_^+^ were determined to have been transported from the Asian continent to Japan as NH_4_NO_3_ and (NH_4_)_2_SO_4_.

Tritium concentration in precipitation after starting the deuterium plasma experiment was within three sigma of the average tritium concentration before the deuterium plasma experiment. There was no clear change in stable isotope composition and chemical species. From this, it seemed that there was no impact from tritium on the surrounding environment of the fusion test facility. The committed effective dose equivalent from drinking water to local residents after the deuterium plasma experiment was calculated to be negligibly small compared with the annual dose limit of 1 mSv. We plan to survey continuously until shutdown of LHD.

## Figures and Tables

**Table 1:** Correlation coefficients of ion species in monthly precipitation collected at Toki, Japan.

	SO_4_^2−^	NO_3_^−^	Na^+^	NH_4_^+^	K^+^	Mg^2+^	Ca^2+^
Cl^−^	0.28	0.21	0.97	0.23	0.10	0.81	0.56
SO_4_^2−^		0.85	0.30	0.80	0.42	0.30	0.56
NO_3_^−^			0.20	0.84	0.26	0.28	0.62
Na^+^				0.21	0.10	0.79	0.54
NH_4_^+^					0.19	0.11	0.60
K^+^						0.04	0.17
Mg^2+^							0.68

## References

[1] Okada S., Momoshima N. (1993). Overview of tritium: Characteristics, sources, and problems. Health Phys..

[2] United Nations Scientific Committee on the Effects of Atomic Radiation (2000). Sources and Effects of Ionizing Radiation, Volume I: Source.

[3] Canadian Nuclear Safety Commission (2009). Investigation of the Environmental Fate of Tritium in the—Part of the Tritium Studies Projec.

[4] United Nations Scientific Committee on the Effects of Atomic Radiation (2010). Sources and Effects of Ionizing Radiation.

[5] Schell W.R., Sauzay G., Payne B.R. (1970). Tritium injection and concentration distribution in the atmosphere. J. Geophys. Res..

[6] Morishima H., Kawai H., Koga T., Niwa T. (1985). The trends of global precipitations. J. Radiat. Res..

[7] Salonen L. (1987). Carbon-14 and tritium in air in Finland after the Chernobyl accident. Radiochim. Acta.

[8] Matsumoto T., Maruoka T., Shimoda G., Obata H., Kagi H., Suzuki K., Yamamoto K., Mitsuguchi T., Hagino K., Tomioka N. (2013). Tritium in Japanese precipitation following the March 2011 Fukushima Daiichi Nuclear Plant accident. Sci. Total Environ..

[9] Nakasone S., Ishimine A., Ishizu Y., Shiroma Y., Tanaka M., Akata N., Kakiuchi H., Sanada T., Furukawa M. (2019). Recent tritium concentration of monthly precipitation in Japan. Radiat. Prot. Dosim..

[10] Patel B., Campling D.C., Macheta P., Sandland K., Schofield P.A. (1999). Health physics aspects of tritium operation at JET. Fusion Eng. Des..

[11] Holtkamp N., for the ITER Project Team (2007). An overview of the ITER project. Fusion Eng. Des..

[12] Cristescu I.R., Cristescu I., Doerr L., Glugla M., Murdoch D. (2007). Tritium inventories and tritium safety design principles for the fuel cycle of ITER. Nuclear Fusion.

[13] Stamoulis K.C., Karamanis D., Ioannides K.G. (2011). Assessment of tritium levels in rivers and precipitation in north-western Greece before the ITER operation. Fusion Eng. Des..

[14] Takeiri Y. (2018). Advanced helical plasma research towards a steady-state fusion reactor by deuterium experiment in large helical device. Atmos.

[15] Tanaka M., Suzuki N., Kato H., Kondo T., Yokosawa M., Kawamata T., Ikeda M., Meguro T., Tanaka T., Sonoi K. (2018). Design and commissioning of exhaust detritiation system for the large helical device. Fusion Eng. Des..

[16] National Institute for Fusion Science (2018). Annual Report for FY 2017 on the Activities of Radiation Safety in LHD Deuterium Plasma Experiment.

[17] Akata N., Kakiuchi H., Tamari T., Tanaka M., Kawano T., Miyake H., Uda T., Nishimura K. (2015). FWT and OBT concentrations in pine needle samples collected at Toki, Japan (1998–2012). Radiat. Prot. Dosim..

[18] Tanaka M., Uda T. (2015). Variation of atmospheric tritium concentration in three chemical forms at Toki, Japan: 2004–12. Radiat. Prot. Dosim..

[19] Akata N., Tanaka M., Kato H., Yamanishi H., Kakiuchi H., Hayashi H., Miyake H., Nishimura K. (2016). Long-term monitoring of tritium concentration in environmental water samples collected at Tono area, Japan. Plasma Fusion Res..

[20] Tanaka M., Akata N., Iwata C. (2019). Environmental tritium around a fusion test facility, Japan. Radiat. Prot. Dosim..

[21] Akata N., Hasegawa H., Sugihara S., Tanaka M., Furukawa M., Kurita N., Kovács T., Shiroma Y., Kakiuchi H. (2019). Tritium, hydrogen and oxygen isotope compositions in monthly precipitation samples collected at Toki, Japan. Radiat. Prot. Dosim..

[22] Japan Meteorological Agency Automated Meteorological Data AcquistionSystem (AMeDAS). http://www.data.jma.go.jp/obd/stats/etrn/index.php.

[23] Akata N., Shiroma Y., Ikemoto N., Kato A., Hegedűs M., Tanaka M., Kakiuchi H., Kovács T. (2018). Atmospheric concentration and deposition flux of cosmogenic beryllium-7 at Toki, central part of Japan. Radiat. Environ. Med..

[24] Japan Chemical Analysis Center Environmental Radioactivity and Radiation in Japan. http://search.kankyo-hoshano.go.jp.

[25] Gusyev M.A., Morgenstern U., Nishihara T., Hayashi T., Akata N., Ichiyanagi K., Sugimoto A., Hasegawa A., Stewart M.K. (2019). Evaluating anthropogenic and environmental tritium effects using precipitation and Hokkaido snowpack at selected coastal locations in Asia. Sci. Total Environ..

[26] Weiss W., Roether W. (1980). The rates of tritium input to the world ocean. Earth Planet. Sci. Lett..

[27] Momoshima N., Okai T., Kaji T., Takashima Y. (1991). Distribution and transformation of various chemical forms of tritium in the environment. Radiochim. Acta.

[28] Akata N., Kakiuchi H., Shima N., Iyogi T., Momoshima N., Hisamatsu S. (2011). Tritium concentrations in the atmospheric environment at Rokkasho, Japan before the final testing of the spent fuel reprocessing plant. J. Environ. Radioact..

[29] Lewis R.R., Fröhlich K., Hebert D. (2008). Contribution to the tritium continental effect. Isot. Environ. Health Stud..

[30] Araguas-Araguas L., Fröehlich K., Rozanski K. (1998). Stable isotope composition of precipitation over southeast Asia. J. Geophys. Res..

[31] Craig H. (1961). Isotope variations in meteoric waters. Science.

[32] Yabusaki S. (2010). Characteristics of stable isotopes in precipitation at Kumagaya City, Saitama Prefecture. Bull. Geo-Environ. Sci..

[33] Dansgaard W. (1964). Stable isotopes in precipitation. Tellus.

[34] Waseda A., Nakai N. (1983). Isotopic compositions of meteoric and surface waters in Central and Northeast Japan. Chikyukagaku.

[35] Tanoue M., Ichiyanagi K. (2016). Deuterium excess in precipitation and water vapor origins over Japan: A review. J. Jpn. Hydrol. Sci..

[36] Hasegawa H., Akata N., Kawabata H., Sato T., Chikuchi Y., Hisamatsu S. (2014). Characteristics of hydrogen and oxygen stable isotope ratios in precipitation collected in a snowfall region, Aomori Prefecture, Japan. Geochem. J..

[37] Ianniello A., Spataro F., Esposito I., Hu M., Zhu T. (2011). Chemical characteristics of inorganic ammonium salts in PM_2.5_ in the atmosphere of Beijing (China). Atmos. Chem. Phys..

[38] Observation Day of Kosa in Gifu Prefecture (1967–2018). http://www.data.jma.go.jp/gmd/env/kosahp/59chiten/632.html.

[39] International Commission on Radiological Protection (2011). ICRP Publication 119; Compendium of Dose Coefficients Based on ICRP Publication 60.

[40] World Health Organization (2008). Guidelines for Drinking-Water Quality, Third Edition Incorporating the First and Second Addenda Volume I; Recommendation.

